# c-Met Targeting Enhances the Effect of Irradiation and Chemical Agents against Malignant Colon Cells Harboring a *KRAS* Mutation

**DOI:** 10.1371/journal.pone.0113186

**Published:** 2014-11-26

**Authors:** Yingbo Li, Jinxi Wang, Xing Gao, Weihua Han, Yongxiang Zheng, Huan Xu, Chuanling Zhang, Qiuchen He, Lihe Zhang, Zhongxin Li, Demin Zhou

**Affiliations:** 1 State Key Laboratory of Natural and Biomimetic Drugs, Peking University, Beijing, China; 2 Second Department of Surgery, the Fourth Hospital of Hebei Medical University, Hebei, China; 3 School of Pharmaceutical Sciences, Peking University, Beijing, China; National Cancer Center, Japan

## Abstract

Although EGFR-targeted therapy has been beneficial to colorectal cancer patients, several studies have showed this clinical benefit was restricted to patients with wild-type *KRAS* exon 2 colorectal cancer. Therefore, it is crucial to explore efficient treatment strategies in patients with *KRAS* mutations. c-Met is an emerging target for the development of therapeutics against colorectal cancer. In this study, we first used the SW620 cell line, which has an activating *KRAS* mutation, to generate a stable cell line with conditional regulation of c-Met, which is an essential gene for growth and an oncogene. Using this approach, we evaluated the benefits of combined c-Met-targeted therapy with irradiation or chemical agents. In this cell line, we observed that the proliferation and migration of SW620 cells were reduced by the induction of c-Met shRNA. Furthermore, c-Met knockdown enhanced the anti-proliferative effects of 5-FU and Taxol but not cisplatin, irinotecan or sorafenib. These enhancements were also observed in another colon cancer cells line HCT-116, which also has a *KRAS* mutation. The response of SW620 cells to irradiation was also enhanced by c-Met knockdown. This method and obtained data might have important implications for exploring the combinatory effects of targeted therapies with conventional medications. Moreover, the data suggested that the combination of c-Met-targeted therapy with chemotherapy or irradiation might be an effective strategy against colorectal cancer harboring a *KRAS* mutation.

## Introduction

Targeted therapy is the most attractive medication that blocks the growth of cancer cells by interfering with specific target molecules that are essential for carcinogenesis and tumor growth [1]. Many targeted therapies have been approved or are currently in clinical trials [2], [3]. Colorectal cancer is the fourth leading cause of cancer-related mortality worldwide. The development of targeted therapies, including anti-EGFR monoclonal antibodies (such as panitumumab and cetuximab), has been beneficial to colorectal cancer patients, and these therapies are becoming standards for treatment of metastatic colorectal cancer. The combination of targeted therapy with chemotherapy also results in an overall survival advantage in patients with advanced disease [4], [5]. Unfortunately, the benefits of panitumumab and cetuximab treatments are restricted to patients with tumors encoding a wild-type *KRAS*. *KRAS* mutation is now considered the crucial biomarker in predicting non-response to EGFR-targeted therapy either as a single agent or in combination with chemotherapy [6], [7]. Because *KRAS* mutation frequently occurs in colorectal cancer patients [8], it is important to explore efficient therapies for patients harboring a *KRAS* mutation.

c-Met belongs to the family of receptor tyrosine kinases whose only known natural ligand is hepatocyte growth factor (HGF) [9], [10]. Aberrant c-Met expression and signaling have been documented in most solid tumors, including colorectal cancer [1], [11], [12]. In addition, high levels of HGF are often detected in the serum of colorectal cancer patients [13], [14], thus generating even more aggressive tumor cells. Therefore, c-Met represents an emerging target for the development of therapeutics against colorectal cancer.

For these reasons, the SW620 human colorectal cancer cell line, which contains an activating *KRAS* (G12V) mutation, was used in the present study. We developed an SW620-shRNA stable cell line in which c-Met, both an essential gene for growth and an oncogene, is conditionally regulated. We evaluated the effect of c-Met targeting alone or c-Met targeting in combination with irradiation or a variety of anticancer drugs on malignant colon cancer cell lines harboring a *KRAS* mutation. These results might have important implications for those who are using combination of targeted therapy with conventional medications to evaluate their potential therapeutic benefit as well as for the development of treatment strategies against colorectal cancer with *KRAS* mutations.

## Materials and Methods

### Reagents

Cisplatin, 5-fluorouracil (5-FU), paclitaxel (Taxol), doxycycline (DOX), SU11274 and HGF were purchased from Sigma Aldrich. PHA-665752 was purchased from Selleckchem. Antibodies against Met, caspase-3, cleaved caspase-3, PARP and actin were purchased from Cell Signaling Technology. The antibody against Ki-67 was obtained from Abcam.

### Cell Culture

Human colon carcinoma cell line SW620, which has a point mutation in codon 12 of *KRAS* gene [15], was purchased from Shanghai Institutes for Biological Sciences. SW620 cells and the derived cell lines with different shRNA-expression cassettes were cultivated at 37°C and 100% air in Leibovitz's L-15 medium (M&C Gene Technology) supplemented with 10% tetracycline-free fetal bovine serum (FBS, Gibco), 100 U/ml penicillin and 100 µg/ml streptomycin. Human colon carcinoma cell line HCT-116, which has a point mutation in codon 13 of *KRAS* proto-oncogene [16], was purchased from ATCC. HCT-116 cells were cultivated at 37°C and 5% CO_2_ in RPMI-1640 medium (M&C Gene Technology) supplemented with 10% FBS (PPA), 100 U/ml penicillin and 100 µg/ml streptomycin.

Human embryonic kidney-293T cells were cultivated at 37°C and 5% CO_2_ in DMEM medium (M&C Gene Technology) supplemented with 10% FBS (PAA), 100 U/ml penicillin and 100 µg/ml streptomycin.

### Construction of siRNA-expressing vectors and transduction of colon cancer cells

The pSD31 lentiviral vector was used for constitutive siRNA expression, and this vector contained a BamHI site downstream of the 5′-LTR. The c-Met-targeting shRNA and scramble shRNA cassettes driven by the hU6 promoter were ligated into pSD31 as previously reported [17]. The inducible pSD400 lentivector was derived from pSD31 by replacing the SV40-PuroR expression cassettes with CMV-TetR-IRES-Puro and introducing a BamHI in the 3′-LTR. Cloning of the inducible shRNA-expressing cassettes into pSD400 was performed as previously reported [17].

### Cell proliferation assay

After treatment, the cells were plated at a density of 4000 cells/well in 96-well plates and incubated in L-15 medium supplemented with or without 400 nM DOX. Cell proliferation was determined using an Alamar blue assay. In the Alamar blue reduction assay, the blue non-fluorescent dye, resazurin, is reduced to pink fluorescent resorufin by mitochondrial enzymes and other enzymes in viable cells. A multilabel counter (Tecan Infinite M200 PRO, Swiss) was used to measure fluorescence at 530 nm excitation and 590 nm emission.

### Transwell migration assay

Cell migration was assessed using a 24-well polycarbonate membrane insert system (8 µM pores; BD Falcon) as described previously [18]. The chambers were filled with 600 µl of medium containing 0.5% FBS and 50 ng/ml HGF. Cells (5×10^5^) were resuspended in 200 µl of medium containing 0.5% FBS and seeded onto the upper side of the insert. The cells were then incubated for 12 h to allow migration through the membrane to the lower side of the insert. After washing with PBS, the cells were fixed in 4% paraformaldehyde. The non-migrating cells on the upper surface of the membrane were removed using cotton swabs. The migrating cells were stained with Hoechst 33342 and observed by fluorescent microscopy.

### Colongenic assay and determination of the survival curve

After treatment, the cells were seeded into 6-well plates at a density of 1500 cells/dish and cultivated for 14 days. The cells were then fixed and stained with crystal violet staining solution. The surviving fraction and dose-survival curves were calculated as previously described [19].

### Western blot analysis

The cells were lysed using RIPA lysis buffer containing 1% PMSF and 1% protease inhibitor cocktail (Roche), and the lysates were subjected to Western blot analysis as described previously [20]. Met (1∶2000), caspase-3 (1∶1000), cleaved caspase-3 (1∶1000), PARP (1∶1000) and actin (1∶2000) were used as primary antibodies.

### Immunofluorescence assay

Immunofluorescence staining was performed as previously described using antibodies against human Met (1∶100) [21]. Hoechst 33342 (5 µg/ml; Beyotime Biotechnology) was used to visualize the nuclei. Stained specimens were visualized using a TCS-SP5 laser-scanning confocal microscope (Leica Microsystems, Germany).

### Real-Time PCR analysis

Total RNA was isolated using TRIzol reagent (Invitrogen) according to the manufacturer's instruction. First-strand cDNA was generated from RNA by reverse transcription followed by real-time PCR using the ABI PRISM 7300 sequence detection system as described previously [22]. The following primers were used for amplification: c-Met, 5′- GAGGTTCACTGCATATTCTCC -3′ (F) and 5′- CACTGATATCGAATGCAATGG -3′ (R); and human actin, 5′-CACCAGGGCTGC TTTTAACTC-3′ (F) and 5′-GATGATGACCCTTTTGGCTCC -3′(R).

### In vivo studies

Animal procedures were approved by the Peking University Health Science Center Institutional Animal Care and Use Committee. SW620 cells (5×10^6^) were injected subcutaneously into the right flank of male BALB/c-nu mice. Treatments were initiated when the tumor volume reached approximately 100 mm^3^. The mice were randomly divided into three treatment groups receiving intraperitoneal (i.p.) injections as follows: 5-FU (40 mg/kg), PHA-665752 (25 mg/kg), or 5-FU (40 mg/kg) plus PHA-665752 (25 mg/kg). The mice in the control group received i.p. injections of physiological saline containing 2.5% DMSO. Tumor sizes were calculated with the following formula: (mm^3^)  =  (π×L×W^2^)/6. Eight nude mice were used in each group.

The immunostaining assay was performed as previously described [23]. Ki-67 (1∶65) was used as the primary antibody. A TUNEL (TdT-mediated dUTP nick end labeling) assay (Roche) was used to detect apoptotic cells in tumor tissues according to the manufacturer's instruction. The slides were counterstained with hematoxylin and imaged using light microscopy.

### Statistical analysis

The data were expressed as the mean ± standard deviation (SD) of at least three independent experiments. The data were subjected to Student's paired two-tailed *t-*test. A P-value <0.05 was considered to be statistically significant.

## Results

### c-Met knockdown via siRNA transient transfection

**Figure 1:**
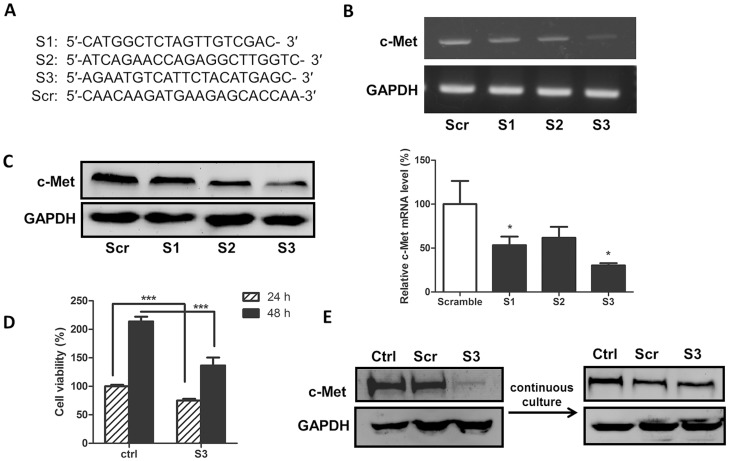
c-Met siRNA transient and stable transfection. (A) Sequences of c-Met-siRNAs and scramble siRNA. (B) and (C) c-Met-siRNAs were transiently transfected into SW620 cells, and the efficiency of c-Met knockdown was determined by (B) Western blot and (C) real-time PCR analyses. (D) SW620 cells were transiently transfected with c-Met-siRNA3. The cell viability was determined with an Alamar blue assay 24 or 48 h post-transfection. (E) The c-Met expression in SW620 (Ctrl), scramble-siRNA (Scr) or c-Met-siRNA3 (S3) stable knockdown cells was analyzed by a Western blot assay. c-Met expression returned to normal levels after the continuous cultivation of c-Met-siRNA3 stable knockdown cells. *P<0.05 and ***P<0.001.

Three c-Met-targeting siRNAs plus a scramble siRNA (Fig. 1A) were designed and transiently transfected into SW620 cells. The efficiency of c-Met knockdown was evaluated by real-time PCR and Western blot analyses. As shown in Fig. 1B, the RT-PCR data demonstrated that the expression level of c-Met mRNA was reduced 72 h post-transfection by all three siRNAs with >70% suppression by siRNA3. Consistently, c-Met expression at the protein level was also significantly reduced by the three siRNAs, and siRNA3 had the strongest reducing effect (Fig. 1C). However, strong cellular toxicity was also observed concomitantly with lipid-mediated transfection, thus resulting in approximately 40% of the transfected cells being unviable 24 and 48 h post-transfection (Fig. 1D).

### c-Met stable knockdown via stable transduction

To address the toxicity issue resulting from transient transfection, we utilized the lentivector system, which usually generates less cellular toxicity during shRNA delivery. The most potent siRNA (siRNA3) and the scramble siRNA were cloned into the pSD31 lentivector driven by the hU6 promoter. Stable transduced cells were generated after one week of puromycin selection after parental cells in a parallel experiment died. Western blot analysis indicated that the levels of c-Met protein in the transduced SW620 cells encoding the c-Met-targeting shRNA3 cassette were remarkably reduced compared to cells encoding the scrambled shRNA cassette (Fig. 1E). However, the transduced cells became unhealthy following continuous culture and grew slowly with the remaining surviving cells displaying no c-Met knockdown (Fig. 1E). These results suggested that long-term silencing of c-Met is not compatible with long-term growth of SW620 and that the generation of stable cells constitutively expressing c-Met-shRNA is not feasible. Therefore, controlling the level of c-Met expression temporally in colon cells via inducible RNAi is essential to generating stable cell lines.

### c-Met conditional knockdown

**Figure 2:**
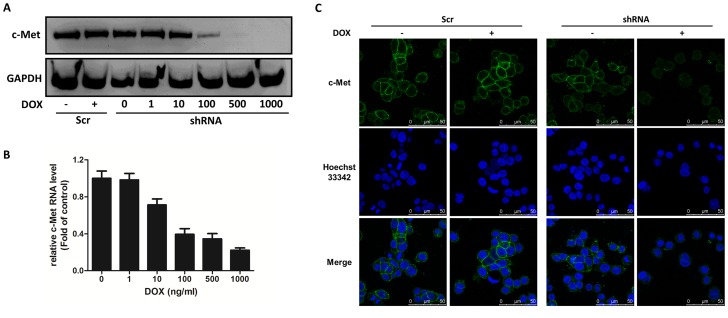
c-Met levels are regulated by DOX in c-Met conditional knockdown SW620 cells. Cells transduced with pSD400 containing scrambled shRNA (Scr) or c-Met-shRNA (shRNA) were treated with DOX for 72 h, and the cells were processed for (A) Western blot analysis, (B) real-time PCR analysis and (C) immunofluorescence staining. The data shown are representative of three independent experiments with similar results.

To generate stable cells in which c-Met can be conditionally knocked down, the shRNA3 sequence was cloned into pSD400, an all-in-one inducible vector for doxycycline-dependent shRNA expression [17], [24]. As illustrated in Fig. 2A, the SW620 cells transduced with pSD400 either containing c-Met-shRNA3 (SW620-shRNA cells) or a scrambled shRNA cassette (SW620-scramble cells) had comparable levels of c-Met protein to the parental cells, thereby indicating that the shRNA was not leaky under non-induced conditions. Adding doxycycline (DOX) to the culture medium led to a significant reduction of c-Met in SW620-shRNA cells at both the protein and mRNA levels. There was no c-Met knockdown in the control SW620-scramble cells regardless of DOX treatment (Fig. 2A). In addition, we observed that the level of c-Met in SW620-shRNA cells was remarkably sensitive to the concentration of DOX, and DOX concentrations greater than 500 ng/ml caused undetectable levels of c-Met (Fig. 2A and 2B). Conditional c-Met knockdown after DOX addition was also confirmed by confocal microscopy as shown in Fig. 2C. c-Met, which is normally observed on the cellular surface (as shown in green), almost completely disappeared after adding 400 nM DOX to the transduced SW620-shRNA cells. There was no obvious change in the transduced SW620-scamble cells after DOX treatment (Fig. 2C). Thus, a stable cell line for the conditional knockdown of the c-Met gene was established allowing the study of the response of SW620 cells in the presence or absence of DOX mimicking the therapeutic consequence of c-Met targeting.

### Effect of c-Met knockdown on tumor cell proliferation and migration in SW620 cells

**Figure 3:**
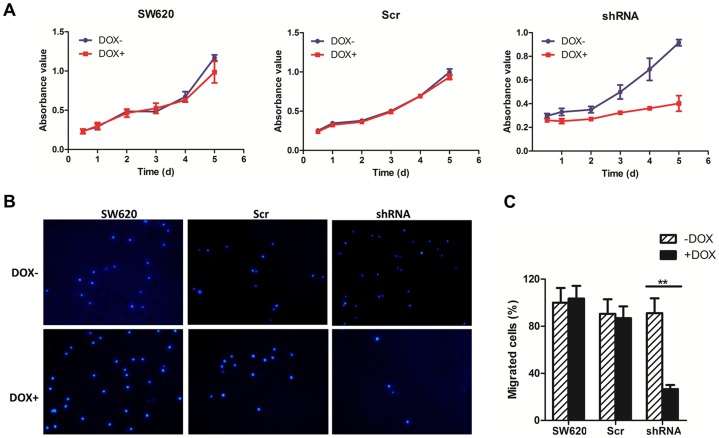
The effects of c-Met conditional knockdown on cell proliferation and migration. (A) Effect of c-Met knockdown on cell proliferation. SW620, SW620-Scr and SW620-shRNA cells were treated with or without DOX (400 nM) for 72 h. The cells were then seeded in 96-well plates and cultivated for 1–5 days. Cell proliferation was determined using an Alamar Blue assay. The values are expressed as the mean ± SD. (B) and (C) Effect of c-Met knockdown on cell migration. SW620, SW620-Scr and SW620-shRNA cells were treated with DOX (400 nM) for 72 h. Cell migration was then determined using a transwell migration assay. (B) Representative results of the migration assay. (C) The number of migrating cells was expressed as a percentage of SW620 cells without DOX treatment. The data are shown as the mean ± SD. **P<0.01.

We next evaluated the effect of c-Met targeting on cell proliferation and migration. Following the conditional knockdown of c-Met by 400 nM DOX, the proliferation of SW620-shRNA cells was significantly inhibited as shown in a cell growth time course. As a control, DOX had no effect on the proliferation of SW620 cells and the derived SW620-scramble cells (Fig. 3A). These data clearly demonstrated that the downregulation of c-Met remarkably decreases the proliferation of SW620 tumor cells. In addition, the effect of c-Met downregulation on cell migration was evaluated using a transwell migration assay. As shown in Fig. 3B, the number of SW620-shRNA cells that migrated to the lower side of the chamber was significantly reduced following c-Met knockdown with DOX addition (P<0.01). Moreover, there was no apparent alteration in either SW620 or SW620-scramble cells regardless of DOX treatment. These data were consistent with the previous conclusion that c-Met is a target for treatment of colon cancer and is also a feasible target in the SW620 colon cancer cell line, which contains an activating *KRAS* mutation. Accordingly, SW620-shRNA was established as an ideal model for evaluating the combinatory effect of c-Met targeting with irradiation or chemical agents.

### The combinatory effect of c-Met targeting and conventional anti-cancer agents in SW620 cells

**Figure 4:**
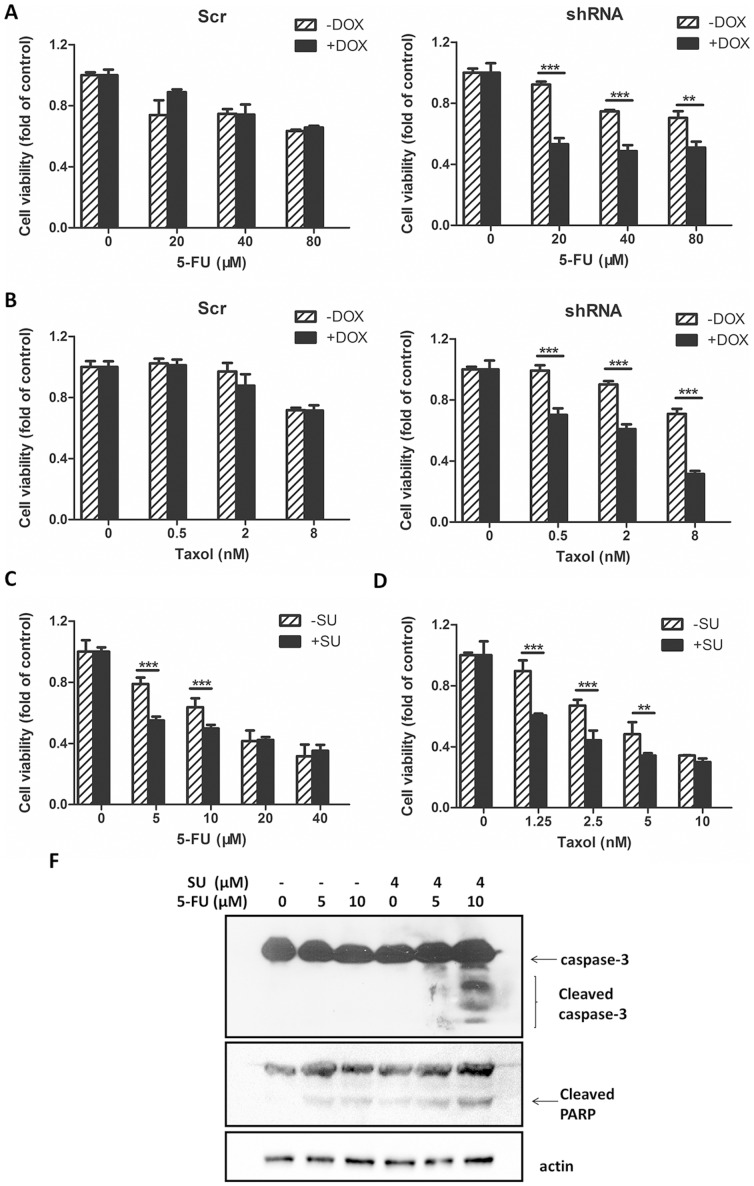
Decreased c-Met enhanced the sensitivity of SW620 cells to 5-FU and Taxol. (A) and (B) c-Met knockdown increased the anti-proliferative effects of 5-FU and Taxol. SW620-Scr and SW620-shRNA cells were treated in the presence or absence of DOX (400 nM) for 72 h and then exposed to different concentrations of (A) 5-FU or (B) Taxol for 48 h. Cell proliferation was determined using an Alamar blue assay. (C) and (D) SU11274 increased the anti-proliferative effects of 5-FU and Taxol. SW620 cells were treated with serial concentrations of (C) 5-FU or (D) Taxol with or without SU11274 (4 µM) for 48 h. Cell proliferation was tested using an Alamar blue assay. (E) SW620 cells were treated with 5-FU (0, 5, or 10 µM) with or without SU11274 (SU; 4 µM) for 48 h, and the cell lysates were then subjected to Western blot analysis to analyze caspase-3 cleavage and PARP cleavage using actin as a loading control. **P<0.01 and ***P<0.001.

Using the SW620-shRNA stable cell line and the SW620-scramble control cell line, we determined the combinatory effects of c-Met targeting and conventional chemotherapeutic agents for colon cancer in cells with mutated *KRAS*. Agents with different anti-cancer mechanisms were selected, including traditionally used agents (5-FU, cisplatin, Taxol, and irinotecan) and a targeted agent (sorafenib). 5-FU displayed a dose-dependent inhibition of proliferation in both SW620-scramble and SW620-shRNA cells, thus underscoring the cellular toxicity of 5-FU as an anti-cancer agent (Fig. 4A). The addition of DOX to the medium of SW620-scramble cells did not enhance the 5-FU-mediated inhibition of proliferation, thereby indicating the low cellular toxicity of DOX. In contrast, the SW620-shRNA cells treated with DOX promoted the inhibition of proliferation caused by 5-FU (Fig. 4A). A similar trend was also observed for Taxol when SW620-shRNA cells were co-treated with Taxol and DOX (Fig. 4B). However, DOX failed to increase the inhibition of proliferation in SW620-shRNA cells caused by cisplatin, irinotecan or sorafenib, thereby indicating that the combination of c-Met targeting with these three individual agents did not lead to an improvement of the treatment (Fig. S1). To confirm the combined effect observed in the inducible cells, SU11274, a specific c-Met inhibitor, was utilized instead of the c-Met knockdown in the proliferation assessment. We found that SU11274 enhanced the anti-proliferative effects of 5-FU and Taxol against SW620 cells (Fig. 4C and 4D), which was consistent with the data obtained from SW620-shRNA cells. The enhancement of the suppressive effect of 5-FU by c-Met targeting might be attributable to apoptosis as evidenced by increased cleavage of caspase-3 and PARP in the SU11275 plus 5-FU groups (Fig. 4E).

### The combinatory effect of c-Met targeting with 5-FU in SW620 xenograft tumor-harbored nude mice

**Figure 5:**
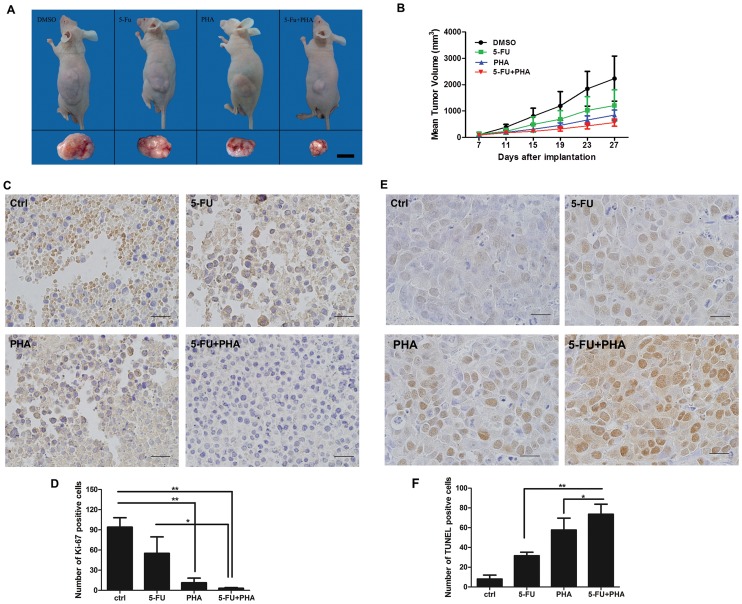
Effects of c-Met knockdown on the antitumor activity of 5-FU chemotherapy in nude mice bearing human colon cancer SW620 xenografts. (A) and (B) Antitumor efficacy in the SW620 model with 5-FU, PHA-665752 (PHA), or 5-FU plus PHA-665752 (5-FU + PHA) treatments. (A) Representative image of the SW620 model. (B) Tumor weight changes in the SW620 model. The data are shown as the mean ± SD (n = 8). (C) and (D) Ki67-positive staining and statistics for cells per field of view from paraffin-embedded sections of SW620 tumors treated with 5-FU, PHA or 5-FU plus PHA. Scale bar  = 20 µm. (E) and (F) TUNEL-positive staining and statistics for cells per field of view from paraffin-embedded sections of SW620 tumors treated with 5-FU, PHA or 5-FU plus PHA. Scale bar  = 20 µm. *P<0.05 and **P<0.01.

We then determined the combinatory effects of 5-FU and c-Met targeting in the SW620 human colon cancer model *in vivo*. PHA-665752 (PHA) is a specific inhibitor of c-Met. As shown in Fig. 5A and 5B, treatment with 5-FU or PHA alone had little inhibitory effects on tumor growth. However, the average tumor size in the 5-FU plus PHA group was significantly decreased compared to the group treated with 5-FU alone, thereby suggesting that c-Met targeting enhanced the inhibitory effects of 5-FU treatment on tumor growth. Moreover, it is important to notice that there was no significant difference in the body weight between the control group and any of the treatment groups indicating the safety of the combinatorial administration of 5-FU and PHA-665752 in this dose range.

We further detected the effects of 5-FU, PHA-665752 or 5-FU plus PHA-665752 on proliferation and apoptosis in tumor tissues. Downregulation of the Ki-67 proliferation marker was observed in the 5-FU and PHA-665752 treatment groups. The combination treatment of 5-FU and PHA-665752 exerted stronger inhibitory effects on the expression of Ki-67 (Fig. 5C and 5D). TUNEL staining was used to detect apoptotic cells in tumor tissues. As shown in Fig. 5E and 5F, treatments of 5-FU or PHA-665752 alone or in combination all significantly increased the number of TUNEL-stained cells compared to the control group. Compared to the groups treated with 5-FU or PHA-665752 only, 5-FU plus PHA-665752 significantly increased the apoptotic cell number (P<0.05), which was consistent with the *in vitro* data that the enhancement of the suppressive effect of 5-FU by c-Met targeting might be attributed to the induction of apoptosis.

### SU11274 enhanced the anti-proliferative effects of 5-FU or Taxol in HCT-116 cells

**Figure 6:**
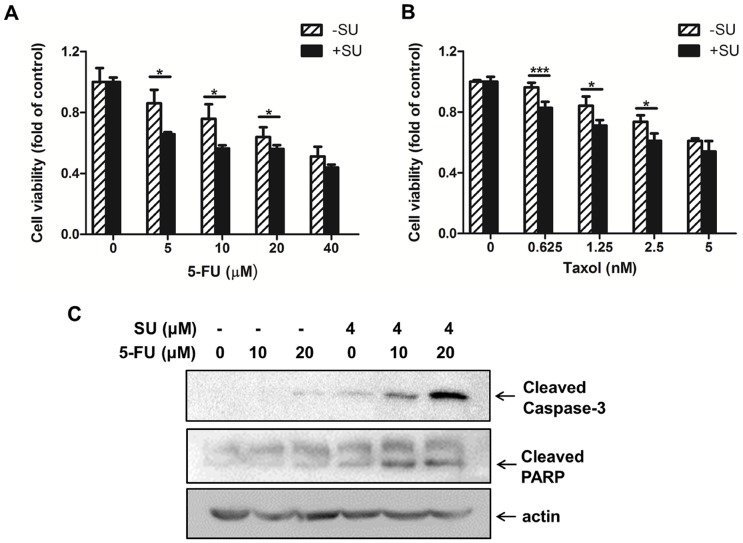
Decreased c-Met enhanced the sensitivity of HCT-116 cells to 5-FU and Taxol. (A) and (B) SU11274 increased the anti-proliferative effects of 5-FU and Taxol. HCT-116 cells were treated with serial concentrations of (A) 5-FU or (B) Taxol with or without SU11274 (4 µM) for 48 h. Cell proliferation was tested using an Alamar blue assay. (C) HCT-116 cells were treated with 5-FU (0, 10, or 20 µM) with or without SU11274 (SU; 4 µM) for 48 h, and the cell lysates were then subjected to Western blot analysis to analyze caspase-3 cleavage and PARP cleavage using actin as a loading control. *P<0.05 and ***P<0.001.

To exclude the possibility that the observed combinatory effects are restricted to SW620 cells, another colorectal carcinoma cell line HCT-116 was used. This cell line also has a point mutation in *KRAS* proto-oncogene. Fig. 6A and 6B showed that addition of the c-Met inhibitor SU11274 enhanced the anti-proliferative effects of 5-FU and Taxol in HCT-116 cells, which was consistent with the data obtained from SW620 cells. Fig. 6C demonstrated that this combination resulted in more apoptotic cells as evidenced by increased cleavage of caspase-3 and PARP in the SU11275 plus 5-FU groups. In addition, no significant enhancement was observed in the combination treatment of SU11274 with cisplatin, irinotecan or sorafenib in HCT-116 cells (Fig. S2).

### The combinatory effect of c-Met targeting and irradiation on SW620 cells

**Figure 7:**
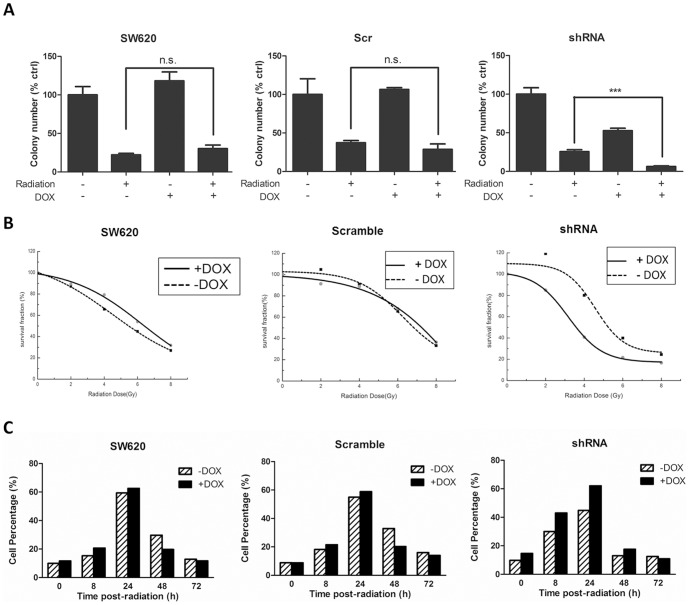
c-Met knockdown results in radiosensitization of SW620 cells. (A) SW620, SW620-Scr and SW620-shRNA cells were treated with or without DOX (400 nM) for 72 h, irradiated by an absorption dose of 4 Gy/min and then subjected to a colongenic assay. (B) SW620, SW620-Scr and SW620-shRNA cells were treated with or without DOX (400 nM) for 72 h and were exposed to different dosages of radiation (0, 2, 4, and 8 Gy). Radiobiological survival curves were obtained based on the colongenic assay. (C) Effect of c-Met knockdown on the percentage of cells arrested in the G2/M phase. The cell cycle distribution was determined by flow cytometry. c-Met knockdown with DOX addition increased the percentage of cells arrested in the G2/M phase 8 and 24 h post-radiation.

Ionizing radiation is one of the most traditional ways to control malignant cell growth in cancer therapy. Combination of c-Met targeting with irradiation might further inhibit the proliferation of colon cancer cells. To test this hypothesis, SW620, SW620-scramble and SW620-shRNA cells were treated with DOX, irradiated by an absorption dose rate of 4 Gy/min, and subjected to a colongenic assay. As shown in Fig. 7A, 4 Gy/min radiation reduced the colony formation ability to approximately 30% in the SW620, SW620-scramble and SW620-shRNA cells compared to the control cells. The addition of DOX in the SW620 or SW620-scramble cells did not further inhibit colony formation. However, in the SW620-shRNA cells, the colony number was reduced to 6.3% with the addition of DOX. A similar trend was also observed in HCT-116 cells. Combination treatment of SU11274 and irradiation significantly reduced the colony formation as compared to irradiation or SU11274 treated alone group (Fig. S3). Furthermore, cells were exposed to different dosages of radiation (0, 2, 4, and 8 Gy), and radiobiological survival curves were obtained as shown in Fig. 7B. These data also indicated that c-Met knockdown resulted in radiosensitization of SW620 cells. To identify the potential mechanism, we performed flow cytometry to monitor cell cycle alteration. Compared to SW620-shRNA cells treated by irradiation alone, the number of cells arrested at the G2/M phase after co-treatment with irradiation and DOX was increased at both 8 and 24 h post-radiation, thereby suggesting that the increased DNA damage may lead to the enhanced sensitivity of cells to c-Met targeting.

## Discussion

RNAi is an important approach to study gene function via specific mRNA degradation, and it is useful to mimic targeted therapy [25]. However, long-term silencing of host genes required for cell growth using constitutive expressed shRNA is not suitable [17], [26]. As shown in this study, constitutive knockdown of c-Met eventually prevented cell survival, thereby making it impossible to evaluate the combinatory effect of c-Met targeting with conventional therapeutic routes. Using an all-in-one inducible RNAi system, we generated an SW620-shRNA stable cell line in which c-Met (both an essential gene for host growth and also an oncogene) could be conditionally regulated. This stable cell line used with the control SW620-scramble cell line provided a suitable platform to evaluate the consequence of c-Met targeting alone or in combination with irradiation or different anticancer drugs in malignant colon cells. The data obtained might have important implications to predict potential therapeutic benefits.

Our experiments demonstrated that targeting c-Met inhibited SW620 cell proliferation and migration, thus supporting the development of c-Met inhibitors and HGF/c-Met antagonists as anti-cancer drugs in colorectal malignancy [27]. More importantly, via the inducible RNAi system, we found that c-Met knockdown enhanced the sensitivity of SW620 cells containing a *KRAS* mutation to 5-FU and Taxol but not to cisplatin, irinotecan and sorafenib. All of these agents are widely used anti-cancer or adjuvant medicines in the treatment of metastatic cancer and non-advanced colorectal cancer. The enhancement of c-Met inhibition on the anti-proliferative effects of 5-FU and Taxol was also observed in another colon cancer cell lines HCT-116 which also containing *KRAS* mutation. We next selected 5-FU as a representative drug to investigate the combinatory effects of 5-FU and c-Met targeting *in vitro* and *in vivo*. The enhancement of 5-FU was confirmed by co-treatment of SW620 cells with 5-FU and SU11274, a well-established c-Met inhibitor (Fig. 4C). Caspase-3, a crucial trigger of apoptosis, was activated when SW620 cells were co-treated with 5-FU and SU11274. 5-FU-induced cleavage of PARP was also increased. Additionally, the animal experiment also suggested that the combined treatment of c-Met targeting and 5-FU generated more effective anti-cancer activity than 5-FU therapy alone. Inhibition of c-Met with its selective inhibitor, PHA-665752, significantly enhanced the inhibitory effect of 5-FU on the growth of SW620 xenograft tumors in immunodeficient mice as exhibited by the decreases in both tumor size and Ki-67 expression in the combinatory treatment group. We also compared apoptosis induced by 5-FU, PHA-665752 or 5-FU plus PHA-665752 treatments by TUNEL staining of the tumor tissue slides. Although 5-FU or PHA-665752 treatment alone increased the number of TUNEL staining cells, the combination of 5-FU and PHA-665752 significantly increased the number of apoptotic cells in the SW620 xenograft tumors. These results were consistent with the *in vitro* data showing that 5-FU plus c-Met targeting more significantly induced apoptosis in SW620 cells. These results suggested that administration of 5-FU in combination with c-Met targeting inhibited the growth of SW620 cells through the induction of apoptosis *in vitro* and *in vivo*. A combination of 5-FU and c-Met targeting might be a feasible strategy in malignant colon cancers with *KRAS* mutations. In addition, we observed that c-Met knockdown sensitized SW620 cells to radiation. Analysis of the cell cycle distribution indicated that a reduction in c-Met increased the number of cells arrested in G2/M phase, therefore generating an increase in genomic damage after irradiation, which may partially explain why c-Met knockdown sensitized SW620-shRNA cells to irradiation compared to SW620 and SW620-scramble cells.

Aiming to develop an approach to evaluate the benefits of a combination of c-Met targeting and irradiation or chemical agents on malignant colon cancer cells with *KRAS* mutations, we generated a stable cell line in which the expression of c-Met (both an essential gene for host growth and also an oncogene) was conditionally regulated. Using this cell line, we found that the proliferation and migration of colon cancer cells were reduced after induction of the shRNA. Furthermore, c-Met knockdown enhanced the anti-proliferative effects of 5-FU and Taxol but not cisplatin, irinotecan and sorafenib in SW620 cells. In addition, we observed that the response of SW620 cells to irradiation was also enhanced by c-Met knockdown. These results might have important implications for patients using a combination of targeted therapy with conventional medications to evaluate their potential therapeutic benefit. Moreover, this work suggests that a combination of c-Met-targeted therapy with chemotherapy and irradiation might be effective strategies to treat colorectal cancer with *KRAS* mutations.

## Supporting Information

Figure S1 **c-Met knockdown fails to enhance the sensitivity of SW620 cells to cisplatin, irinotecan or sorafenib.** SW620, SW620-Scr and SW620-shRNA cells were cultured in the presence or absence of DOX (400 nM) for 72 h and exposed to different concentrations of cisplatin (A), irinotecan (B), or sorafenib (C) for 48 h. Cell proliferation inhibition was determined by an Alamar blue assay.
(TIF)

Figure S2 **SU11274 treatment fails to enhance the sensitivity of HCT-116 cells to cisplatin, irinotecan or sorafenib.** HCT-116 cells were exposed to different concentrations of cisplatin (A), irinotecan (B), or sorafenib (C) in the presence or absence of SU11274 (4 µM) for 48 h, then cell viability was determined by an Alamar blue assay.
(TIF)

Figure S3 **SU11274 enhances the irradiation-induced inhibition on HCT-116 cell survival.** HCT-116 cells were exposed to SU11274 (4 µM) for 24 h, and then irradiated by an absorption dose of 4 Gy/min. The effects of SU11274 and irradiation on cell survival were determined by clonogenic assay.
(TIF)
