# Supplementary figures and images for: c-Met Targeting Enhances the Effect of Irradiation and Chemical Agents against Malignant Colon Cells Harboring a KRAS Mutation
Source: PLoS One. 2014 Nov 26;9(11):e113186. doi: 10.1371/journal.pone.0113186 (PMC4245100; doi:10.1371/journal.pone.0113186)

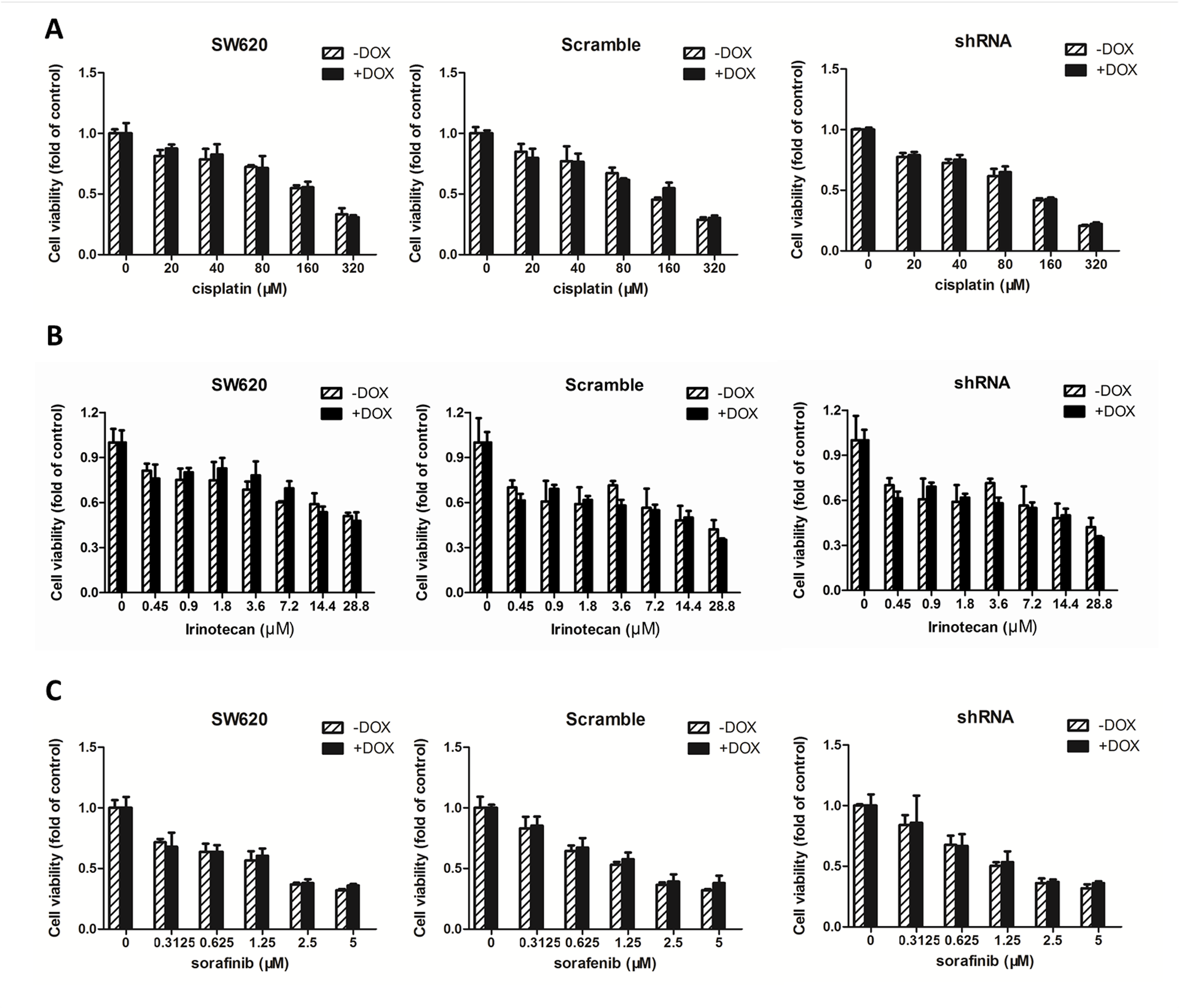

Supplement: Figure S1 — c-Met knockdown fails to enhance the sensitivity of SW620 cells to cisplatin, irinotecan or sorafenib. SW620, SW620-Scr and SW620-shRNA cells were cultured in the presence or absence of DOX (400 nM) for 72 h and exposed to different concentrations of cisplatin (A), irinotecan (B), or sorafenib (C) for 48 h. Cell proliferation inhibition was determined by an Alamar blue assay. (TIF) [file pone.0113186.s001.tif]

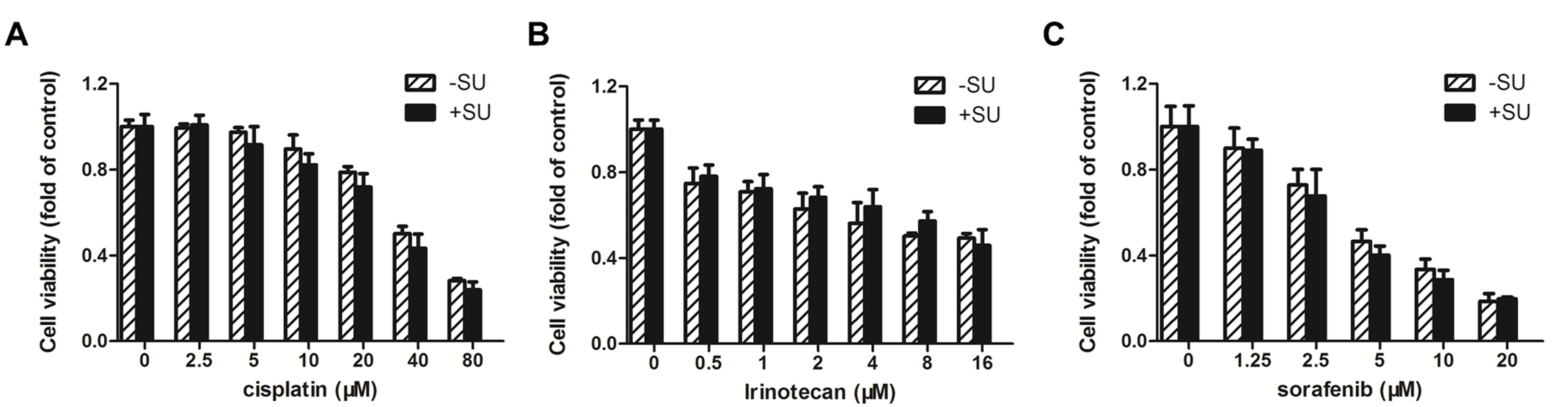

Supplement: Figure S2 — SU11274 treatment fails to enhance the sensitivity of HCT-116 cells to cisplatin, irinotecan or sorafenib. HCT-116 cells were exposed to different concentrations of cisplatin (A), irinotecan (B), or sorafenib (C) in the presence or absence of SU11274 (4 µM) for 48 h, then cell viability was determined by an Alamar blue assay. (TIF) [file pone.0113186.s002.tif]

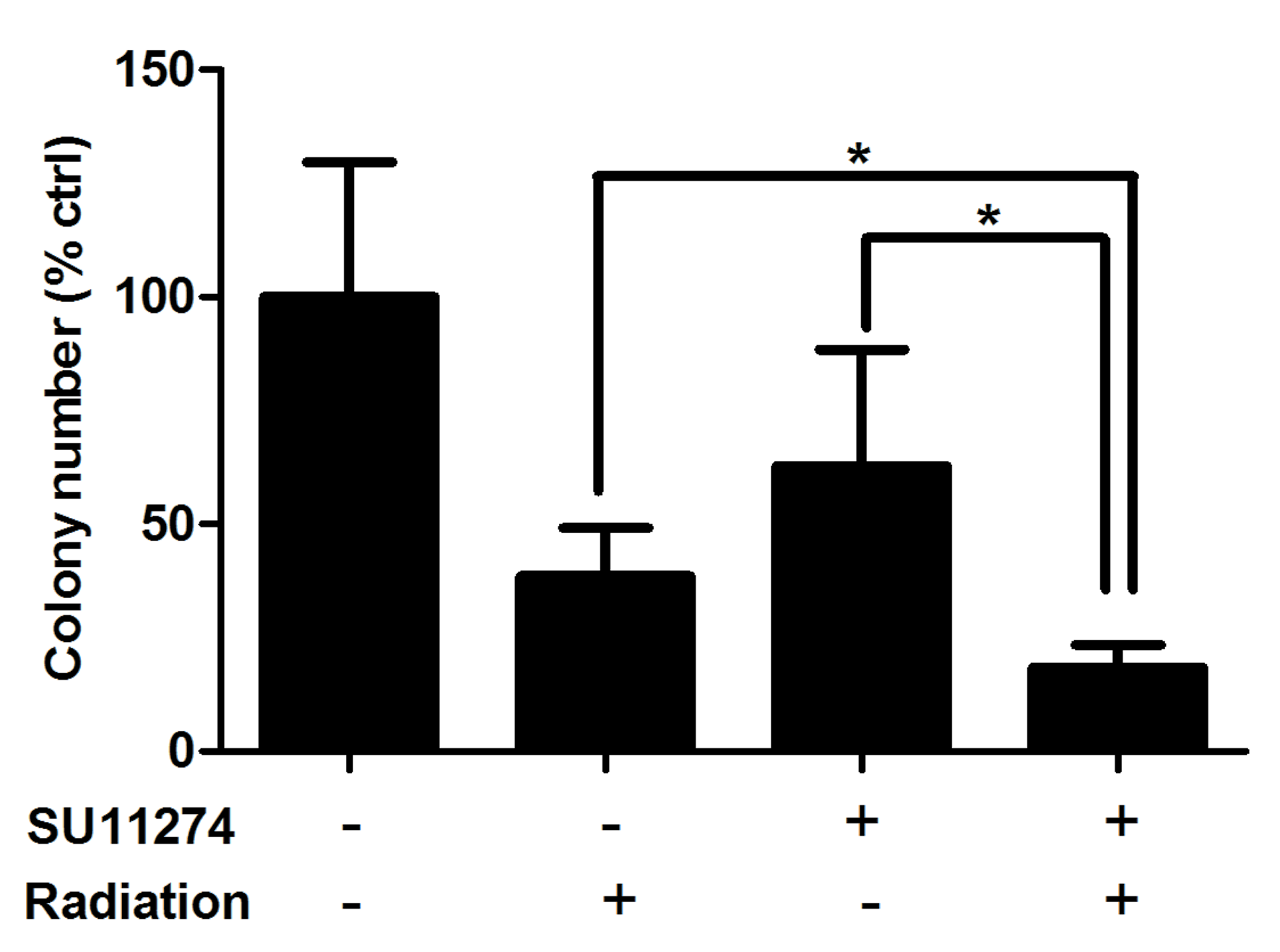

Supplement: Figure S3 — SU11274 enhances the irradiation-induced inhibition on HCT-116 cell survival. HCT-116 cells were exposed to SU11274 (4 µM) for 24 h, and then irradiated by an absorption dose of 4 Gy/min. The effects of SU11274 and irradiation on cell survival were determined by clonogenic assay. (TIF) [file pone.0113186.s003.tif]
